# Increased Hydrogen Production by Genetic Engineering of *Escherichia coli*

**DOI:** 10.1371/journal.pone.0004432

**Published:** 2009-02-12

**Authors:** Zhanmin Fan, Ling Yuan, Ranjini Chatterjee

**Affiliations:** 1 Department of Plant and Soil Sciences and Kentucky Tobacco Research and Development Center, University of Kentucky, Lexington, Kentucky, United States of America; 2 Farasis Energy, Inc., Hayward, California, United States of America; Baylor College of Medicine, United States of America

## Abstract

*Escherichia coli* is capable of producing hydrogen under anaerobic growth conditions. Formate is converted to hydrogen in the fermenting cell by the formate hydrogenlyase enzyme system. The specific hydrogen yield from glucose was improved by the modification of transcriptional regulators and metabolic enzymes involved in the dissimilation of pyruvate and formate. The engineered *E. coli* strains ZF1 (Δ*focA*; disrupted in a formate transporter gene) and ZF3 (Δ*narL*; disrupted in a global transcriptional regulator gene) produced 14.9, and 14.4 µmols of hydrogen/mg of dry cell weight, respectively, compared to 9.8 µmols of hydrogen/mg of dry cell weight generated by wild-type *E. coli* strain W3110. The molar yield of hydrogen for strain ZF3 was 0.96 mols of hydrogen/mol of glucose, compared to 0.54 mols of hydrogen/mol of glucose for the wild-type *E. coli* strain. The expression of the global transcriptional regulator protein FNR at levels above natural abundance had a synergistic effect on increasing the hydrogen yield in the Δ*focA* genetic background. The modification of global transcriptional regulators to modulate the expression of multiple operons required for the biosynthesis of formate hydrogenlyase represents a practical approach to improve hydrogen production.

## Introduction

The present emphasis on the diversification of global energy sources, while reducing the consumption of fossil fuels, has placed renewed interest on hydrogen as an alternative fuel and its synthesis through microbe-mediated bioconversions. Microbes have evolved unique mechanisms for hydrogen generation, and some of these mechanisms are being explored for biotechnological applications including nitrogenase-mediated and fermentative hydrogen production [1]. Efforts to improve hydrogen production have been focused on pathway redirection, identification and engineering of oxygen-tolerant hydrogenases, improvement in hydrogen molar yields and development of efficient hydrogen separation techniques from bioreactor headspace [1]–[5].

**Figure 1:**
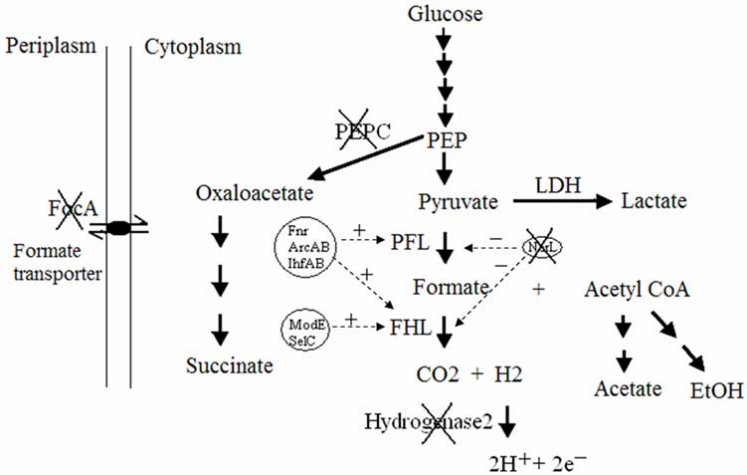
The genetic modification of metabolic pathways and regulatory components for hydrogen production in *E. coli*. The metabolic flows are indicated by solid arrows. Some key enzyme systems are labeled. The key global regulators and their regulatory targets are circled and indicated by dashed arrows, respectively. Pluses (+) represent activation, and minuses (−) represent repression. Crosses (X) indicate chromosomal gene disruptions.

The production of hydrogen by facultative anaerobic organisms such as *E. coli* is a characteristic of mixed acid fermentation. Under anaerobic conditions, the fermentation products comprise a mixture of ethanol, succinate, lactate, acetate, and formate (Fig. 1). Succinate is produced via the succinate pathway. A key reaction in this pathway is carboxylation of phosphoenolpyruvate (PEP) to the four-carbon compound oxaloacetate by phosphoenolpyruvate carboxylase (PEPC) [6]. PEP is also converted to pyruvate which is subsequently converted to acetyl-CoA and formate by pyruvate formate lyase (PFL), and to lactate by lactate dehydrogenase (LDH). Formate hydrogenlyase (FHL) catalyzes the conversion of formate to carbon dioxide and hydrogen [7], [8]. The intracellular level of formate is determined by the rates of biosynthesis and metabolism of formate, and also by formate transporter FocA which is a membrane protein involved in formate transport into and out of the cell [9]. Knockout of the *focA* gene results in intracellular accumulation of formate [10]. The FHL enzyme complex is comprised of formate dehydrogenase H (FDH_H_), and a hydrogen-evolving hydrogenase (hydrogenase 3) [11], [12]. The biosynthesis of PFL and FHL are up-regulated by the action of multiple transcriptional regulators including the global transcriptional factors Fnr, ArcAB and integration host factor (IHF) [13]–[15], and repressed by the dual transcriptional regulator NarL (Table 1)[16]. The transcription of the *fhl* regulon is regulated by the primary and secondary transcriptional activator FHLA and ModE [17], [18]. The expression of *fhlA* is activated by Fnr in response to the cellular redox state [19], [20]. The biosynthesis of FDH_H_ also requires the expression of *selC* gene which encodes a tRNA for the incorporation of selenocysteine to FDH_H_ (Table 1) [8], [11], [21].

**Table 1:** Genes engineered in this study, and their functions.

Gene	Function of gene product
*focA*	Transmembrane protein involved in formate transport.
*hybC*	Large catalytic subunit of hydrogenase 2; catalyzes dihydrogen oxidation.
*narL*	Global transcriptional regulator of anaerobic metabolism; represses the transcription of *nik* operon genes, *fdhF* and *pflB* genes; activates the transcription of formate dehydrogenase N, which couples formate oxidation to nitrate reduction.
*ppc*	Phosphoenolpyruvate carboxylase (PEPC) catalyzes the carboxylation of phosphoenolpyruvate (PEP) to oxaloacetate (OAA).
*fnr*	Global transcriptional regulator of anaerobic metabolism; activator of *fhlA* and *pflB* genes, and of the *hyp*, *nik* and *moa* operon genes.
*arcAB*	Global transcriptional regulator of aerobic and anaerobic metabolism; activator of *pflB*, and repressor of the *hyb* and *hya* operon genes.
*ihfAB*	Global transcriptional regulator activating the *pflB* and *fhlA* genes, and genes of the *hyc* and *hyp* operons.
*modE*	Secondary transcriptional activator of the *fhl* regulon.
*selC*	tRNA for selenocysteine incorporation into FDH-F polypeptide of FHL.

Successful efforts to redirect pyruvate towards hydrogen in *E. coli*, have involved the up-regulation of the FHL complex and the disruption of pathways competing for pyruvate [4], [22]–[24]. It is estimated that over 50 genes distributed across at least 20 distinct genetic loci define fermentative hydrogen metabolism in *E. coli* [8]. In this study, our strategy involves the systematic modification of multiple, discrete, metablic segments that include global regulatory, transport, and auxiliary components required for FHL biosynthesis, processing and assembly. The modification of key regulatory elements represents a practical strategy for the coordinated engineering of genes and operons that perform distinct biochemical functions related to the production of hydrogen. This approach has the potential to achieve the balance of cofactor and precursor supply pathways with the biosynthesis of structural polypeptides without placing an unnecessary metabolic burden on the cells [25]. Here, we describe increases in specific and molar yields of hydrogen achieved by the modification of the *focA*, *ppc*, *narL*, and *fnr* genes involved in precursor transport and metabolism, and the global regulation of fermentative hydrogen production by *E. coli*.

## Results and Discussion

### Strategy for genetic modification

The fermentation pathways initiate by the non-oxidative cleavage of pyruvate to acetyl-CoA and formate by PFL; formate is subsequently metabolized to hydrogen and carbon dioxide by FHL. Our strategy for improving fermentative hydrogen production involved the modification of precursor transport and metabolism, and of regulatory elements, to direct the flow of the two key precursor metabolites pyruvate and formate towards hydrogen, and achieve increased expression of both structural and auxiliary components of the FHL enzyme system. The biochemical reactions and regulatory elements that were modified by the inactivation of specific chromosomal genes, or by plasmid-directed gene over-expression are illustrated schematically in Figure 1; the functions of the corresponding gene products are summarized in Table 1.

The inactivation of the four selected genes in the chromosome of *E. coli* strain W3110 was hypothesized to increase the cellular concentrations of pyruvate and formate, reduce the re-oxidation of evolved hydrogen, and relieve the repression of genes required for PFL and FHL biosynthesis. The *ppc* gene encoding phosphoenolpyruvate carboxylase (PEPC) was disrupted to increase cellular pyruvate concentrations available for formate biosynthesis [6], [7]. The gene encoding the formate transporter, *focA*, was inactivated to elevate intra-cellular formate levels for FHL biosynthesis and conversion to hydrogen [10]. *E. coli* synthesizes two uptake hydrogenases, hydrogenase1 and 2, which oxidize hydrogen to protons with the release of electrons. In this study the *hybC* gene, which encodes the large catalytic subunit of hydrogenase 2, was inactivated to reduce the oxidation of hydrogen [26]. The global transcriptional regulator NarL represses transcription of the structural genes of FHL (*fdhF*), PFL (*pflB*), and of the genes of the *nik* operon, which are required for the transport and metabolism of nickel for Hydrogenase cofactor biosynthesis [16]. To eliminate the repression described, the *narL* gene was inactivated.

The global transcriptional regulators Fnr, ArcAB and IhfAB activate multiple genes of anaerobic pyruvate and formate metabolism. Targets for these global regulators include *fhlA* (the specific transcriptional activator of the *fhl* regulon), *pflB*, and the genes of the *moa*, *nik* and *hyp* operons required for the biosynthesis of the molybdopterin and Ni-Fe cofactors of FHL (Table 1) [15], [19], [27], [28]. The effects on hydrogen yield of multi-copy expression of the global regulators *fnr*, *arcAB* and *lhfAB*, as well as *ModE* (the secondary transcriptional activator of the *fhl* regulon), and the tRNA for selenocysteine incorporation (*selC*), were examined in wild-type W3110 and Δ*focA* (ZF1) strains.

### Increased hydrogen yields by gene-inactivated *E. coli* strains

**Figure 2:**
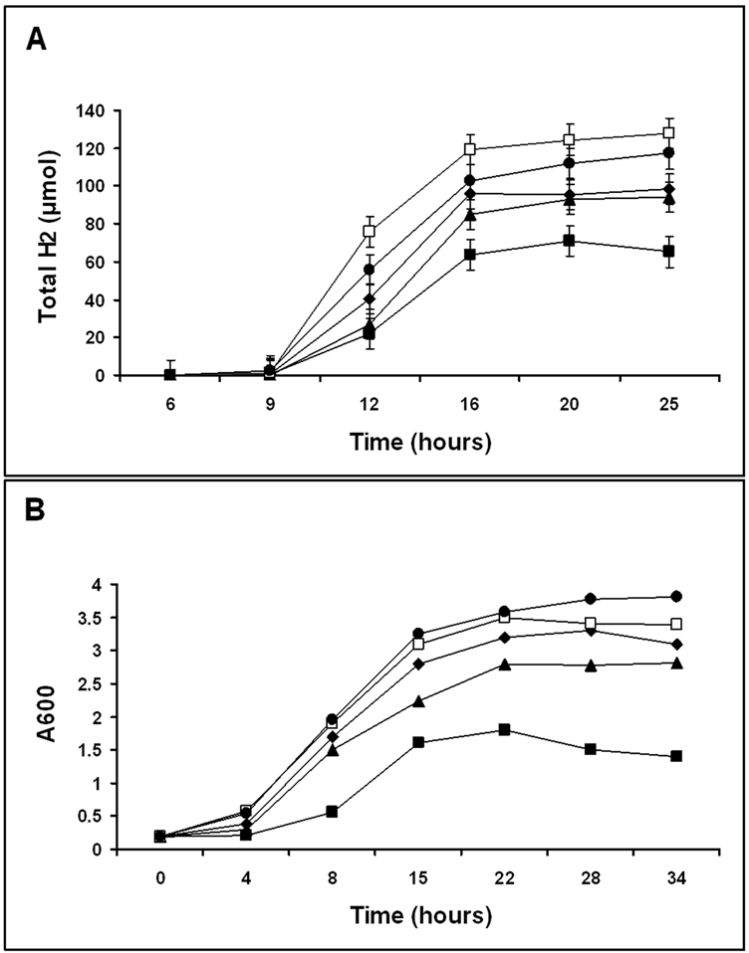
Hydrogen production and anaerobic growth rates of WT and single gene inactivated *E. coli* strains. Hydrogen production rate (A, µmol of H_2_) and anaerobic growth rate (B, A600) by strains W3110 (⧫), ZF1 (▪), ZF2 (▴), ZF3 (□), ZF4 (•) on M9 medium supplemented with 1% (w/v) glucose.

The rate of hydrogen production and of growth by wild-type strain W3110, and four strains containing specific chromosomal gene disruptions were examined (Figures 2A and 2B). The maximum rate of hydrogen generation occurred between 9 and 16 hours following inoculation of M9 medium under the experimental conditions described. Strains ZF3 (W3110Δ*narL*) and ZF4 (W3110Δ*ppc*) exhibited the highest rates of hydrogen production, and had accumulated about 20% to 30% more hydrogen in the culture headspace than W3110 in 25 hours (Figure 2A). Strain ZF1 (W3110Δ*focA*) exhibited a slightly slower rate of hydrogen generation than W3110 and accumulated 33% less hydrogen by the end of the fermentation experiment. Strains ZF2 (W3110Δ*hybC*) and W3110 had comparable rates of hydrogen formation to each other. The slower rate of hydrogen generation observed in the later stages of fermentation correlated with an increased accumulation of biomass, possibly resulting from the hydrogen oxidizing activity of uptake hydrogenases 1, 2, and/or 4, generating protons and electrons for cellular ATP synthesis, or by inhibition of hydrogenase 3 by the evolved hydrogen that accumulated in culture headspace [8], [26]. The complete consumption of glucose accompanied by reduced glycolytic flux to pyruvate and subsequently to formate could account for the simultaneous reductions in growth and H_2_ production rates. Strains W3110, ZF3 (W3110Δ*narL*) and ZF4 (W3110Δ*ppc*) exhibited similar generation times and reached similar final cell densities. Strain ZF2 (W3110Δ*hybC*) grew at a slightly slower rate than W3110, while strain ZF1 (W3110Δ*focA*) grew at a slower rate than W3110 and reached a 50% lower final culture density (Figure 2B). The lower growth rates of strains ZF1 and ZF2 might result from the increased acidification of cellular cytoplasm from the disruption of formate export, and by the decrease in cellular ATP synthesis by the disruption of an uptake hydrogenase, respectively. The rate of anaerobic growth and of hydrogen production by the strains in TYP medium followed a profile similar to that seen in M9-glucose medium, with the highest rates of hydrogen production occurring between 4 and 7 hours of fermentation. The culture generation times were increased by about 2.5-fold in TYP medium, with A_600_ values reaching about 6.5 by 10 hours (data not shown).

The specific hydrogen yields were determined for W3110 and the gene-inactivated strains at 17 hours following inoculation of M9-glucose medium (Table 2). All four gene-inactivated strains exhibited 14% to 50% higher specific hydrogen yields compared to W3110. Strains ZF1 (W3110Δ*focA*) and ZF3 (W3110Δ*narL*) had specific hydrogen yields of approximately 14 µmols/mg dry cell mass, representing an increase of about 46% over the yield by W3110. The increased hydrogen yield by strain ZF1 (W3110Δ*focA*) is consistent with the hypothesis that an increase in the level of intracellular formate can either lead to enhanced synthesis of the FHL polypeptides via the transcriptional activator FhlA, or elevate the available substrate pool for FHL [29], [30]. The negative effect on growth rates by the *focA* deletion might be alleviated if the intracellular formate can be efficiently metabolized by FHL (further described below). The 50% increase in specific hydrogen yield by strain ZF3 (W3110Δ*narL*) showed that the elimination of transcriptional repression of *nik* operon, and of *fdhF* and *pflB* genes by the NarL, results in the increased pyruvate metabolism towards hydrogen [8], [31].

**Table 2:** Hydrogen yields for strain W3110 with or without defined chromosomal gene disruptions.

Strains	Hydrogen yield (μmol/mg dry cell mass)	Hydrogen yield from glucose (mol/mol glucose)
W3110	9.80±0.72	0.54±0.04
ZF1 (W3310Δ*focA*)	14.85±0.83	0.63±0.04
ZF2 (W3310Δ*hybC*)	12.14±0.88	0.70±0.05
ZF3 (W3310Δ*narL*)	14.39±0.90	0.96±0.07
ZF4 (W3310Δ*ppC*)	11.20±0.89	0.73±0.05
ZF5 (W3310Δ*focA*Δ*hybC*)	8.65±0.78	Not determined

The hydrogen yields were determined following 17 hours of anaerobic growth on M9 medium containing 415 µmols of glucose.

The specific hydrogen yield of strain ZF2 (W3110Δ*hybC*) was 22% higher than W3110 (Table 2). Of the three *E. coli* uptake hydrogenases, hydrogenase 2 is believed to be the more active one based on *in vitro* kinetic studies [8]. The double mutant ZF5 (W3110Δ*focA*Δ*hybC*) did not produce more hydrogen than the single mutants ZF1 (W3110Δ*focA*) and ZF2 (W3110Δ*hybC*) for reasons that remain unclear. Inactivation of the metabolic enzyme PEPC resulted in approximately 14% increase in specific hydrogen yields.

If all of the pyruvate is directed towards the PFL reaction the theoretical maximum yield of hydrogen from a hexose sugar is 2 mols of H_2_/mol glucose [7]. Strain ZF3 (W3110Δ*narL*) also displayed the best molar yield of hydrogen (mols of H_2_/mol of glucose) of approximately 0.96 compared to 0.54 for W3110 (Table 2). The utility of eliminating transcriptional repression of multiple operons in anaerobic metabolism is highlighted by the effects of the *narL* gene deletion. This deletion confers a 1.5-fold increase in specific hydrogen yield and a 2-fold increase in molar hydrogen yield, while retaining the growth characteristics of the wild-type strain W3110.

To examine the partitioning of glucose in the two high hydrogen yielding strains the distribution of fermentation products of strains ZF1 (W3110Δ*focA*) and ZF3 (W3110Δ*narL*) were compared to that of W3110 (Table 3). These two strains carried out a balanced fermentation, with over 90% of the added glucose converted to products. No remaining glucose was detected in the medium by HPLC and spectrophotometric assays. Medium from strain ZF1 (W3110Δ*focA*) contained 40% less formate compared to strains W3110 and ZF3 (W3110Δ*narL*), consistent with less formate being secreted from the cell due to the disruption of the channel for formate. Lactate formation was increased by about 37% in strain ZF1 (W3110Δ*focA*) compared to W3110, perhaps from the efficient induction of *ldhA* due to lower intra-cellular pH from formate accumulation, or by the inhibition of PFL activity and/or biosynthesis by formate. The formation of ethanol was lowered by 17% and 33% in strains ZF1 (W3110Δ*focA*) and ZF3 (W3110Δ*narL*), respectively, compared to that of W3110. These two strains accumulated succinate and acetate to levels similar to those of strain W3110.

**Table 3:** The fermentation products of strains W3110, ZF1 (W3110Δ*focA*) and ZF3 (W3110Δ*narL*).

	W3110	ZF1 (W3310Δ*focA*)	ZF3 (W3310Δ*narL*)
Hydrogen (µmol)	91.0±1.34	122.84±1.89	118.0±1.42
Formate (µmol)	114.24±1.32	66.86±0.87	112.83±1.26
Lactate (µmol)	158.92±1.47	218.40±1.88	159.66±1.21
Acetate (µmol)	106.36±1.17	107.96±1.15	109.14±1.15
Succinate (µmol)	41.48±0.76	42.85±0.72	43.46±0.81
Ethanol (µmol)	344.0±2.87	285.43±2.25	229.0±1.87

The concentrations of metabolites in culture media were determined following 34 hours of anaerobic growth on M9 medium containing 830 µmols of glucose.

### The effect of multi-copy gene expression on hydrogen yields

Recent reports have described improvements in fermentative hydrogen yields in *E. coli* through the modification of the specific transcriptional activator, and repressor proteins of the FHL system, FhlA and HycA, respectively [22]. The three global regulators Fnr, ArcAB and IhfAB modulate the expression of multiple genes involved in the anaerobic metabolism of pyruvate in *E. coli* [9], [15], [19], [27], [28]. The impact on fermentative hydrogen yields by the over-production of the Fnr, ArcAB, and IhfAB proteins, and of ModE which is the secondary transcriptional activator of the *hyc* operon, was examined in strains W3110 and ZF1 (W3110Δ*focA*). Strain ZF1 (W3110Δ*focA*) was selected because an increased level of cellular formate, combined with the over-expression of formate-metabolizing genes might have a synergistic effect on hydrogen formation. The specific hydrogen yields following induction of the plasmid-bearing strains are shown in Table 4. Strain ZF7, over-expressing of the *fnr* gene in W3110 background, exhibited an approximately 5.5-fold increase in specific hydrogen yield compared to the control strain ZF6 (containing the plasmid without promoter-driven gene expression). Over-expression of the *fnr* gene in the Δ*focA* genetic background (strain ZF13) resulted in the generation of 10 µmol of H_2_/mg of dry cell mass compared to 6 µmol of H_2_/mg of dry cell mass obtained with over-expression of the *fnr* gene in the wild-type W3110 background (strain ZF7) in TYP medium. When induced in M9-glucose medium for 8 hours, strains ZF7 and ZF13 accumulated approximately 24 µmol of H_2_/mg of dry cell mass and 34 µmol of H_2_/mg of dry cell mass, respectively (data not shown). Over-expression of the global regulators ArcAB and IhfAB, and of the secondary transcriptional activator ModE, had no discernible effects on hydrogen yields in either W3110 or ZF1 strains in TYP or in M9-glucose medium. The synergistic effect on increased hydrogen yield by the over-expression of Fnr in the Δ*focA* genetic background suggests that genetic modifications to simultaneously increase both substrate and enzyme levels for hydrogen production are desirable to prevent the accumulation of formate inside the cell. These results show that specific genetic backgrounds provide additive effects on hydrogen yield when combined with increased gene dosage of particular metabolic enzymes or regulatory components.

**Table 4:** Hydrogen yields for strain W3110 and ZF1 (W3110Δ*focA*) when expressing plasmid-borne genes.

Strain	Genotype	Hydrogen yield (μmol/mg dry cell mass)
ZF6	W3110/pACYC177L	1.07±0.03
ZF7	W3110/pfnr	6.23±0.45
ZF8	W3110/parcAB	1.11±0.04
ZF9	W3110/pihfAB	1.14±0.04
ZF10	W3110/pmodE	1.27±0.06
ZF11	W3110/pselC	1.81±0.06
ZF12	W3110Δ*focA*/pACYC177L	0.00±0.00
ZF13	W3110Δ*focA*/pfnr	10.32±0.58
ZF14	W3110Δ*focA*/parcAB	0.19±0.02
ZF15	W3110Δ*focA*/pihfAB	0.61±0.03
ZF16	W3110Δ*focA*/pmodE	0.63±0.03
ZF17	W3110Δ*focA*/pselC	1.19±0.06

The specific hydrogen yields were determined following 4 hours of induction on TYP medium. TYP medium was supplemented with glucose at a final concentration of 0.5% (w/v) for this experiment.

While the Fnr protein activates transcription of operons involved in nickel and molybdenum metabolism for FHL biosynthesis [32], it has not been shown to affect the transcription of genes involved in selenium metabolism. To determine whether selenium metabolism is limiting to hydrogen production, the *selC* gene, encoding the tRNA for selenocysteine incorporation into the FDH_H_ polypeptide, was over-expressed in strains W3110 and ZF1 (W3110Δ*focA*). An approximately 63% increase in hydrogen yield was observed in strain ZF11 which expresses multiple copies of the *selC* gene compared to the negative control, strain ZF6 (Table 4). These results illustrate that hydrogen yields can be increased by improving the efficiency of a pathway for cofactor production for the FHL enzyme system.

One approach to obtaining sustained increases in fermentative hydrogen-yields will be to direct and improve the metabolism of pyruvate and formate towards hydrogen under anaerobic conditions. Genetic modifications that have led to increased hydrogen yields in *E. coli* have thus far focused on the disruption of key metabolic genes of the mixed acid fermentation pathways of *E. coli*, and of specific regulators of the FHL system. This report represents the first example of increased hydrogen production achieved through the modification of two global transcriptional regulators, a cofactor biosynthetic gene, and a transporter for formate. The construction of *E. coli* strains with specific chromosomal gene deletions, have revealed three unique genetic backgrounds that improve fermentative hydrogen production: inactivation of the *focA*, *narL*, and *ppc* genes lead to increased hydrogen yields over that of wild-type *E. coli*. The metabolism of selenium is one of several metabolic pathways required for the processing of metal ions for FHL biosynthesis. The improvement in hydrogen yield observed by the increased expression of the tRNA for selenocysteine suggests that cofactor biosynthesis pathways could be useful targets for modification. The increased hydrogen yields obtained by over-expression of the *fnr* gene, indicates that the anaerobic metabolism of pyruvate and formate can be coordinately up-regulated for improved flux towards hydrogen.

The results presented in this work illustrate the utility of modifying transport, regulatory, and auxiliary pathways related to FHL biosynthesis and function, to achieve increased hydrogen yields. The future application of genome engineering formats in combination with global gene expression analysis to *E. coli* will enable the identification of novel genes and metabolic functions underlying the improved hydrogen producing phenotype [25], [33]. Similar approaches might be extended to enteric microbes that contain the FHL enzyme system to improve hydrogen production from alternate carbon sources, and for the simultaneous production of value-added chemical entities derived from the anaerobic metabolism of pyruvate.

## Materials and Methods

### Genetic manipulations

All strains and plasmids used in this study are listed in Table 5, and PCR primers used are shown in Table 6. The disruption of chromosomal genes in *E. coli* K-12 strain W3110 was performed by the method of Datsenko and Wanner [34]. Plasmid pKD13 was used as the template for amplification of the FRT-flanked Km^R^ gene with primers containing 44 to 50 nucleotide homology extensions. Plasmids pKD46 and pCP20 were used for the replacement of the target gene with the Km^R^ gene, and for subsequent Km^R^ gene removal, respectively. Primer pairs, *focA*-H2P4/*focA*-H1P1, *hybC*-H2P4/*hybC*-H1P1, *nar*-H2P4/*nar*-H1P1, and *ppc*-H2P4/*ppc*-H1P1 were used for the disruption of the *focA*, *hybC*, *narL*, and *ppc* genes, respectively. The entire coding sequences of the *hybC*, *narL*, and *ppc* genes were deleted, while only 589 nucleotides at the 5′ terminal sequence of the *focA* gene were deleted since the sequence downstream of it overlaps the *pfl* operon. The gene disruptions were verified by PCR analyses of genomic DNA using the primers k1 and k2 to the Km^R^ gene, and the FW and RV primers to the flanking sequences of the disrupted genes.

**Table 5:** *E. coli* strains and plasmids used in this study.

Strains and plasmids	Genotype or description	Reference or source
Strains		
W3110	F-l-IN(*rrnD-rrnE*)1 *rph-1*	CGSC# 4470
ZF1	W3110Δ*focA*	This work
ZF2	W3110Δ*hybC*	This work
ZF3	W3110Δ*narL*	This work
ZF4	W3110Δ*ppc*	This work
ZF5	W3110Δ*focA*Δ*hybC*	This work
ZF6	W3110 containing pACYC177L	This work
ZF7	W3110 containing pfnr	This work
ZF8	W3110 containing parcAB	This work
ZF9	W3110 containing pihfAB	This work
ZF10	W3110 containing pmodE	This work
ZF11	W3110 containing selC	This work
ZF12	ZF1containing pACYC177L	This work
ZF13	ZF1 containing pfnr	This work
ZF14	ZF1 containing parcAB	This work
ZF15	ZF1 containing pihfAB	This work
ZF16	ZF1 containing pmodE	This work
ZF17	ZF1 containing pselC	This work
Plasmids		
pfnr	pACYC with LacZ promoter and W3110 *fnr* gene	This work
parcAB	pACYC with LacZ promoter and W3110 *arcA* and *arcB* genes	This work
pihfAB	pACYC with LacZ promoter and W3110 *ihfA* and *ihfB* genes	This work
pmodE	pACYC with LacZ promoter and W3110 *ihfA* and *ihfB* genes	This work
pselC	pACYC with LacZ promoter and W3110 *selC* gene	This work

**Table 6:** Primers used in this study.

Primer	Sequence	Source
*focA*-H2P4	5′-CTTTGTTAGTATCTCGTCGCCGACTTAATAAAGAGAGAGTTAGTATTCCGGGGATCCGTCGACC-3′	This work
*focA*-H1P1	5′-GATACTGTGCTCAAAACCGCTGGCAACAAACATCGCGACCGGCAGTGTAGGCTGGAGCTGCTTC-3′	This work
*focA*-FW	5′-GCCAGGCGAGATATGATCTA-3′	This work
*focA*-RV	5′-CTGCGTACTTCGACAACCAT-3′	This work
*hybC*-H2P4	5′-CCTTTAAAACAAAACGATCATAATCGTCATGAGGCGAGCAAAGCATGAGCATTCCGGGGATCCGTCGACC-3′	This work
*hybC*-H1P1	5′-ATCGGTCAGCAAAATATTGCCGACCCCTAAGACTAAAATACGCATTACAGGTGTAGGCTGGAGCTGCTTC-3′	This work
*hybC*-FW	5′-GTCAGCACTGAGCGCACTGT-3′	This work
*hybC*-RV	5′-CAACGCCGGAACTTCTTCAT-3′	This work
*nar*-H2P4	5′-CACATTCATTAAGGTTATTGCTCATTTAAAGCCTGAAGGAAGAGGTTTACATTCCGGGGATCCGTCGACC-3′	This work
*nar*-H1P1	5′-TTCGCAGCATCGGGTGATCGTCAATCAGCAGGATAGTAGCCGGTTCCTGAGTGTAGGCTGGAGCTGCTTC-3′	This work
*nar*-FW	5′-GCCAATAGCCGAGAATACTG-3′	This work
*nar*-RV	5′-GACTCCGCCAGTTCAATACC-3′	This work
*ppc*-H2P4	5′-CGTGAAGGATACAGGGCTATCAAACGATAAGATGGGGTGTCTGGGGTAATATTCCGGGGATCCGTCGACC-3′	This work
*ppc*-H1P1	5′-ATTTCAGAAAACCCTCGCGCAAAAGCACGAGGGTTTGCAGAAGAGGAAGAGTGTAGGCTGGAGCTGCTTC-3′	This work
*ppc*-FW	5′-CGCATCTTATCCGACCTACA-3′	This work
*ppc*-RV	5′-TGGCCTGTAGCAGAGTAGAG-3′	This work
Plac-*Bam*H-FW	5′-ACATGGATCCGCGCCCAATACGCAAAC-3′	This work
Plac-*Pst*I-RV	5′-ACATCTGCAGAGCTGTTTCCTGTGTGAAAT-3′	This work
Plac-*Sca*I-RV	5′-ACATAGTACTAGCTGTTTCCTGTGTGAAAT-3′	This work
*Fnr*-FW	5′-ACTACTGCAGATGATCCCGGAAAAGCGAATTA-3′	This work
*Fnr*-RV	5′-TCTATGCGCATCAGGCAACGTTACGCGTA-3′	This work
*modE*-FW	5′-ACTACTGCAGATGCAGGCCGAAATCCTTC-3′	This work
*modE*-RV	5′-TCTATGCGCATTAGCACAGCGTGGCGATA-3′	This work
*selC*-FW	5′-ACTACTGCAGATACGCTGCGTGTATTAGGC-3′	This work
*selC*-RV	5′-TCTATGCGCACTTAGACCAGGTATTCCTGAAG-3′	This work
*arcB*-*Xba*I-FW	5′-TCTAGATAAGGCCGCGGAGGATTACACTATGATGAAGCAAATTCGTCTGCT-3′	This work
*arcB*-*Bgl*I-RV	5′-ATCTAGCCGGAAGGGCTCATTTTTTAGTGGCTTTTGCC-3′	This work
*arcA*-FW	5′-ATGCAGACCCCGCACATTCT-3′	This work
*arcA*-*Xba*I-RV	5′-ACTATCTAGATTAATCTTCCAGATCACCGCA-3′	This work
*arcA*-*Xba*I-RV	5′-ACTATCTAGATTAATCTTCCAGATCACCGCA-3′	This work
*ihfB*-*Xba*I-FW	5′-TCTAGATAAGGCCGCGGAGGATTACACTATGATGACCAAGTCAGAATTGAT-3′	This work
*ihfB*-*Bgl*I-RV	5′-ATCTAGCCGGAAGGGCTTAACCGTAAATATTGGCGC-3′	This work
*ihfA*-FW	5′-ATGGCGCTTACAAAAGCTGA-3′	This work
*ihfA*-*Xba*I-RV	5′-ACTATCTAGATTACTCGTCTTTGGGCGA-3′	This work

The plasmid pACYC177 (Invitrogen) was used for the multi-copy expression of the *E. coli* genes *fnr*, *arcAB*, *ihfAB*, *modE*, and *selC*. The genes were amplified by PCR, using Pfx50™ DNA polymerase (Invitrogen) to produce blunt ended PCR products, from genomic DNA of *E. coli* strain W3110. For the construction of pfnr, pmodE and pselC, the *lacZ* promoter sequence from the pGEM®-T easy vector (Promega) was transferred into pAYCY177 by amplification using primers Plac-*BamH*I-FW and Plac-*Pst*I-RV, digestion with *BamH*I and *Pst*I, and ligation into the corresponding sites of pACYC177. The modified pACYC177 vector was designated pACYC177L. The *fnr*, *modE*, and *selC* genes were amplified with primer pairs: *fnr*-FW/*fnr*-RV, *modE*-FW/*modE*-RV and *selC*-FW/*selC*-RV, respectively, digested with *Pst*I and *Fsp*I, and ligated into the corresponding sites of pACYC177L. For the construction of parcAB and pihfAB, primer Plac-*Sca*I-RV was used instead of primer Plac-*Pst*I-RV for the amplification of the *lacZ* promotor, and *Sca*I was used for digestion of the PCR fragment. This modified pACYC177 vector was designated as pACYC177L1. The *arcB* and *ihfB* genes were amplified using primer pairs *arcB*-*Xba*I-FW/*arcB*-*Bgl*I-RV and *ihfB*-*Xba*I-FW/*ihfB*-*Bgl*I-RV, respectively. The two forward primers contained a 40-nucleotide linker sequence from genomic DNA of W3110. The PCR products were digested with *Bgl*I, and ligated to the *Fsp*I/*Bgl*I site of pACYC177L1. Finally, the *arcA* and *ihfA* genes were amplified using primer pairs *arcA*-FW/*arcA*-*Xba*I-RV and *ihfA*-FW/*ihfA*-*Xba*I-RV, digested with *Xba*I, and ligated to *Sca*I/*Xba*I site of pACYC177L1.

### Bacterial strains, media, and growth conditions

All strains of *E. coli* used in this study are listed in Table 5. *E. coli* strains were routinely cultured at 37°C in either phosphate-buffered rich medium TYP (10 g/L tryptone, 5 g/L yeast extract, 12 g/L K_2_HPO_4_, 3 g/L KH_2_PO_4_; pH 7.6) or in M9 salts medium (Sigma-Aldrich) containing trace elements [35], [36]. When required, kanamycin was added at a concentration of 30 µg/ml. For studies requiring plasmid-directed gene expression, 1 mM IPTG was added to the cultures 1 hour following inoculation of the fermentation medium, and induction was performed for approximately 4 hours.

Fermentations were carried out in 37 ml serum vials (Wheaton) containing 15 ml of TYP or M9 medium. Following the addition of medium, the vials were sealed using silicone stoppers and aluminum crimp seals (Supelco), and autoclaved. Prior to inoculation, the headspace was evacuated and flushed with Argon (ultra high purity, Airgas). Supplements included in TYP or M9 medium were: 0.5% or 1% (w/v) glucose; 1 µM sodium molybdate; 1 µM sodium selenite; 50 µM nickel sulfate. Approximately 1.3% (v/v) of inoculum culture, prepared under aerobic conditions in TYP, was used to inoculate fermentation medium. Specific hydrogen yields were determined at approximately 5 hours, and 17 hours in TYP and M9 medium, respectively. Fermentations in 10 ml of M9 medium containing 0.5% glucose (w/v) were run for 17 hours to determine the molar yields of hydrogen. The concentrations of all products generated from glucose were determined for strains cultured anaerobically for 34 hours in 15 ml of M9 medium containing 1.0% glucose (w/v) to allow adequate time for the consumption of all of the glucose contained in the medium. All experiments were repeated six times with at least duplicate fermentations contained within each experiment. The *A*_600_ for strains cultured anaerobically in M9 medium was measured by removing samples anoxically with a syringe in intervals for 34 hours.

### Analytical methods

The gas mixture from culture headspace was withdrawn using gastight syringes (Hamilton), and monitored for hydrogen formation. Hydrogen was detected using a Shimadzu GC-17A gas chromatograph (GC) equipped with a thermal conductivity detector (TCD), a 60/80 Molecular Sieve 5A column (Supelco), with argon as the carrier gas. The Clarity software package (DataApex) was used to analyze the chromatograms.

The formation of fermentation products were detected by high performance liquid chromatography (HPLC) using an Aminex HPX-87H ion exchange column (Bio-Rad; 7.8×300 mm) and a Varian ProStar 410 liquid chromatography instrument equipped with UV absorbance and refractive index detectors. Culture supernatant was filtered through 0.45 µM cellulose acetate filters, and 40 µL of sample was injected. The column was eluted isocratically with 5 mM H_2_SO_4_ at a flow rate of 0.5 ml/minute. Data was analyzed using the Star Chromatography Workstation software. Ethanol was quantified using the Quantichrome assay kit (BioAssay Systems). Glucose was quantified by both HPLC (described above), and by the glucose oxidase (GO) assay kit (Sigma).

Dry cell mass was determined by filtration of the entire culture through a 0.8 µM nitrocellulose membrane, drying the membrane at 60°C in a vacuum oven for about 14 hours, and determining the weight of the membrane.
